# Synergistic activity of a KL51-depolymerase and a *Sugarlandvirus* bacteriophage against ST16 *Klebsiella pneumoniae*

**DOI:** 10.1128/spectrum.02142-25

**Published:** 2025-10-27

**Authors:** Mélanie Roch, Roberto Sierra, Gaël Panis, Willames B. Martins, Diego Andrey

**Affiliations:** 1 Department of Microbiology and Molecular Medicine, Medical School, University of Geneva 218785, Geneva, Switzerland; 2 Division of Infectious Diseases, Department of Medicine, Geneva University Hospitals and Medical School, Geneva, Switzerland; 3 Biomedical Sciences, Faculty of Medicine, University of Geneva 28535 , Geneva, Switzerland; 4 Biology Department, University of Oxford 6396, Oxford, United Kingdom; 5 Ineos Oxford Institute for Antimicrobial Research (IOI), Oxford, United Kingdom; University of California San Diego, La Jolla, California, USA

**Keywords:** bacteriophage, depolymerase, *Klebsiella pneumoniae*

## Abstract

The rising prevalence of multidrug-resistant *Klebsiella pneumoniae* strains, particularly the emerging high-risk ST16 lineage, highlights the urgent need for alternative therapeutic strategies. A major barrier to effective bacteriophage (phage) therapy against *K. pneumoniae* is its polysaccharide capsule, which hinders phage access to surface receptors. In this study, we identified and characterized an anti-KL51 capsule depolymerase derived from the anti-ST16 phage PWKp9B. The recombinant DpoK51-9B enzyme was expressed in *Escherichia coli*, purified, and shown to degrade the KL51 capsule using Percoll density gradient, halo formation on double-layered-agar, and FITC-dextran exclusion assays. The depolymerase exhibited strong activity at nanomolar concentrations, retained function up to 50°C, and was active only against KL51-positive strains, including ST16 and ST231 isolates, but not against strains with other capsule types. Importantly, the depolymerase enabled the lytic activity of the *Sugarlandvirus* phage GPH82, normally inactive against encapsulated ST16, demonstrating potent phage-depolymerase synergy. These findings highlight the potential of recombinant depolymerases to expand phage host range by unmasking the phage receptors on the surface of the bacteria and therefore enhance the efficacy of phages against encapsulated bacteria.

**IMPORTANCE:**

Bacteriophage therapy and phage-derived therapeutics are attracting increasing attention as promising alternatives or adjuvants to antibiotics in the treatment of multidrug-resistant bacterial infections. Among phage-encoded enzymes, depolymerases are of particular interest due to their ability to degrade the bacterial polysaccharide capsule that prevents phage adsorption. Our results highlight the utility of combining recombinant depolymerases with phages to broaden their host range and enhance their activity against encapsulated, drug-resistant pathogens.

## INTRODUCTION

Antimicrobial resistance is one of the most pressing global health challenges of the 21st century (1), contributing to increasing treatment failures, morbidity, and mortality worldwide (2). Among the pathogens that contribute to the antimicrobial resistance crisis, *Klebsiella pneumoniae* stands out as a major human pathogen and a member of the notorious ESKAPE group (3). The burden of *K. pneumoniae* infections is exacerbated by the spread of so-called high-risk clones, lineages that are particularly successful at acquiring and maintaining multidrug resistance determinants while also spreading efficiently in healthcare environments (4). In 2019, multidrug-resistant *K. pneumoniae* was estimated to be responsible for 193,000 excess deaths per year (2).

Besides the widespread clonal complex (CC) 258, additional multidrug-resistant lineages are emerging (3). Of particular concern is the sequence type (ST) 16, which has been increasingly reported in countries such as Brazil, Vietnam, China, and Thailand and is now considered an emerging global threat (5–10) and has gathered attention due to its association with high virulence and multidrug resistance (11). Compared to CC258, ST16 is associated with a higher risk of fatal outcomes (6). This enhanced virulence was coupled with multidrug resistance: ST16 was shown to harbor multiple carbapenemase genes, 16S rRNA methyltransferase, and determinants conferring resistance to colistin (5, 6, 12). The convergence of high virulence and extensive drug resistance underscores the urgent need for novel therapeutic strategies against *K. pneumoniae*.

In this context, bacteriophage (phage) therapy is gaining renewed interest as an alternative or adjuvant treatment option to antibiotics for tackling multidrug-resistant bacterial infections (13, 14). Phages are viruses that infect bacteria, with remarkable specificity determined by both phage traits (e.g., receptor-binding proteins) and bacterial factors (e.g., surface receptors, defense systems) (15). In encapsulated pathogens like *K. pneumoniae*, the capsule, a polysaccharide outer layer, plays a key role in determining phage susceptibility (16–19). This capsule can act both as a barrier, blocking access to surface receptors, and as a receptor itself for certain capsule-targeting phages. The capsule is also a major virulence factor that forms a protective barrier against environmental stressors and the host’s immune defenses (17).

To bypass this defense system, some phages encode tail spike proteins with capsule depolymerase activity, which are enzymes that degrade capsular polysaccharides and expose underlying structures used as receptors by phages (20, 21). Depolymerases are typically highly specific, recognizing only one or a few capsular types with similar structures. This poses a major challenge in *K. pneumoniae*, where over 150 distinct genetic capsular (KL) types have been described (22, 23), while the repertoire of characterized depolymerases remains limited (24). A large part of depolymerase research has focused on identifying depolymerases against different capsule types, K2 and K64 being the most represented in the literature (20, 25). Structural studies of anti-K2 enzymes provided detailed insights into depolymerase trimerization and substrate recognition (26, 27). Since the capsule is a major virulence factor protecting bacteria against the host immune system, depolymerases are investigated as antivirulence agents and have been shown to sensitize bacteria to immune clearance (28, 29). Interestingly, non-encapsulated *K. pneumoniae* strains have also been shown to be susceptible to a broader range of phages (30). This observation suggests a potential strategy: combining capsule-degrading depolymerases with phages to extend their host range.

In this study, we report the identification and characterization of a recombinant depolymerase targeting the KL51 capsule type, commonly associated with the high-risk ST16 clone. We show that this depolymerase synergizes, in a cooperative manner, with the *Sugarlandvirus* bacteriophage GPH82, otherwise inactive against encapsulated ST16 strains. This combinatorial approach illustrates a promising avenue for expanding phage host range and combating encapsulated, drug-resistant *K. pneumoniae*.

## MATERIALS AND METHODS

### *K. pneumoniae* bacterial strains

*K. pneumoniae* clinical isolates harboring the capsule locus (KL) KL51 used for the study are presented in Table 1. A collection of clinical isolates available in the laboratory and harboring other KL was used as controls. Capsule loci were identified using the Kaptive tool or its integrated version in Kleborate (23, 31, 32). Expression of the capsule was verified by Percoll density gradient (see method below).

**TABLE 1:** Activity of phage PWKp9B and its recombinant depolymerase DpoK51-9B on *K. pneumoniae* strains used for the study^*c*^^,^^*d*^

Strain name	ST	KL	Capsule expression	Depolymerase Activity DpoK51-9B^*a*^	Phage PWKp9B lytic activity^*b*^	Origin	Reference
Kpn3	ST16	51	+	+	+	Brazil	Martins et al. (33)
HSP83	ST16	51	+	+	+	Brazil	Andrey et al. (6)
P20	ST16	51	+	+	+	Brazil	Andrey et al. (6)
P31	ST16	51	–	–	+	Brazil	Andrey et al. (6)
VS17	ST16	51	+	+	–	Switzerland	Sierra et al. (9)
CNR48	ST16	51	+	+	–	France	Hennequin et al. (7)
ST231A	ST231	51	+	+	–	Pakistan	Hassan et al. (34)
ST231B	ST231	51	+	+	–	Pakistan	Hassan et al. (34)

^*a*^ DpoK51-9B depolymerase activity assessed by Percoll density gradient and halo on double layered agar.

^*b*^ PWKp9B activity assessed by the formation of lytic plaque on double layered agar.

^*c*^ ”+”, positive.

^*d*^ "-”, negative.

### Bacteriophages

The four bacteriophages infecting *K. pneumoniae* used in this study are described in Table 2: PWKp9B, PWKp5, PWKp1, and GPH82. Anti-ST16 phages PWKp9B, PWKp5, and PWKp5 were previously described by Martins et al. (33). Phage GPH82 was isolated from Geneva (Switzerland) wastewater as part of the GENeva PHage (GENPH) collection project (35) on the host strain HSP79 (ST437, KL64, KPC-2-producing *K. pneumoniae* [6]).

**TABLE 2:** Bacteriophages used in this study

Phage name	Classification	Genome size	Genome accession	Origin	Reference
PWKp9B	*Caudoviricetes, Autoscriptoviridae, Slopekvirinae, Drulisvirus*	44,881 bp	MZ634343.1	Brazil	(33)
PWKp1	*Caudoviricetes, Autoscriptoviridae, Slopekvirinae, Drulisvirus*	44,602 bp	MZ634338.1	Brazil	(33)
PWKp5	*Caudoviricetes, Ackermannviridae, Taipeivirus*	157,530 bp	MZ634341.1	Brazil	(33)
GPH82	*Caudoviricetes, Demerecviridae, Sugarland virus*	111,710 bp	SAMN49946575	Switzerland	This study

### Media and growth conditions

*K. pneumoniae* isolates and *E. coli* laboratory strains were grown in Luria Bertani (Miller formula) broth with 180 rpm shaking or on LB agar plates at 37°C, otherwise indicated. For phage infection experiments, buffered LB containing 50 mM Tris-HCl pH 7.4, 8 mM MgSO_4_, and 3 mM CaCl_2_, together with 5 g/L agar when soft agar media was needed.

### Identification of depolymerase

The depolymerase was identified from the phage genome of pWkp9B (accession number MZ634343.1) using the phage depolymerase finder tool (available at bit.ly/phagedpo) (36). The putative depolymerase was then analyzed with InterPro to identify the key functional domains (37).

### Cloning and purification of DpoK51-9B

**Fig 1:**
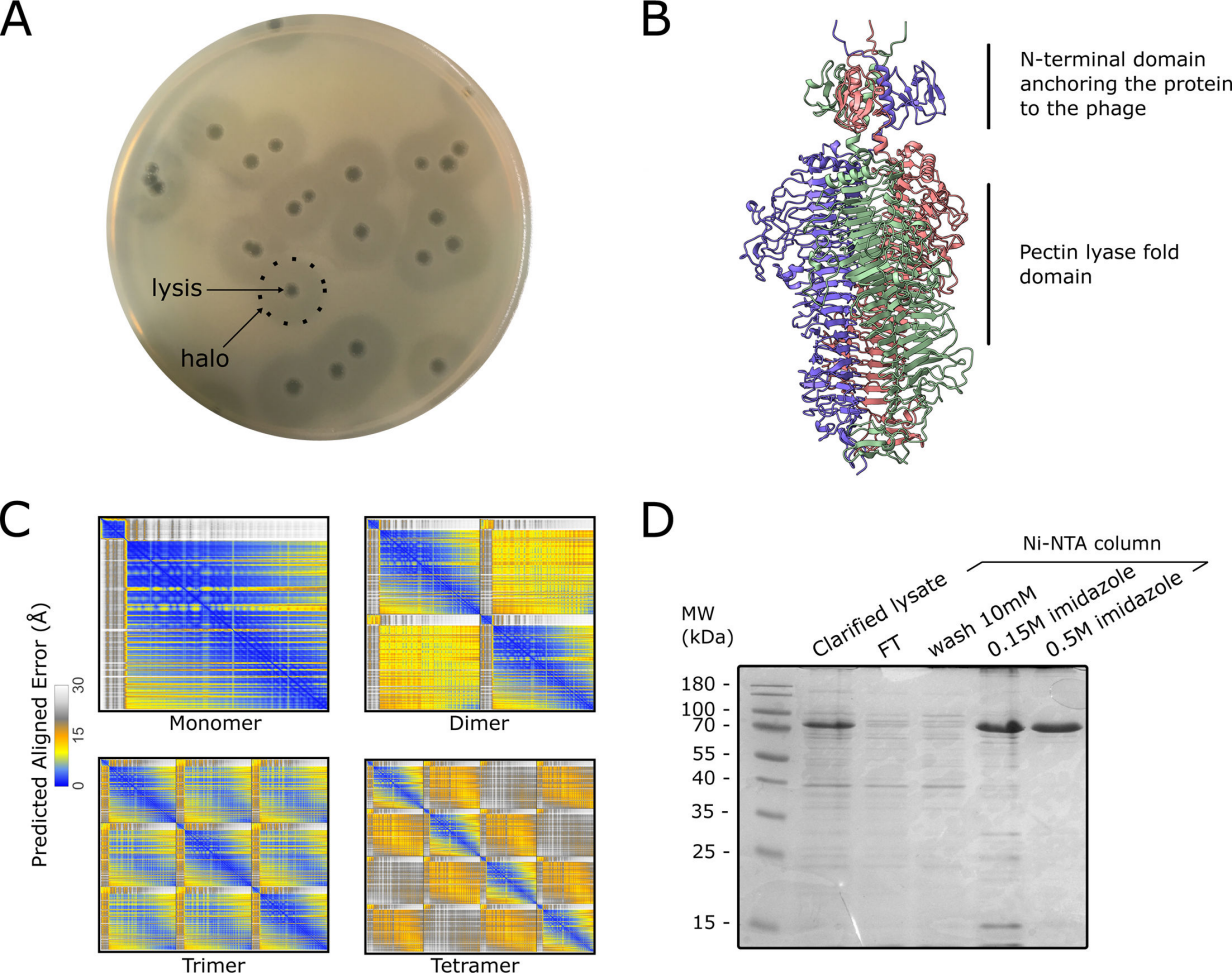
Identification and purification of the KL51 depolymerase DpoK51-9B (**A**) Plaque morphology of phage pWKp9B on *K. pneumoniae* ST16 strain HSP83, displaying clear lytic plaques with large surrounding halos indicative of depolymerase activity. (**B**) SDS-PAGE gel stained with Coomassie Brilliant Blue showing the purity of the recombinant DpoK51-9B at different purification steps. (**C**) AlphaFold-predicted structure of the depolymerase, modeled as a trimer with domains predicted from InterPro. (**D**) Predicted aligned errors (PAE) of the depolymerase DpoK51-9B modeled as a monomer, dimer, trimer, and tetramer, indicating optimal confidence in the trimeric form.

The candidate depolymerase gene was cloned in pET24a with a C-terminus-6xHis-tag using the primers listed in Table 3. The sequence of the insert was verified by Sanger sequencing. Plasmids were then transformed into *Escherichia coli* Tuner (λDE3) (Novagen). Expression of the recombinant protein was induced at OD 0.5–0.8 with 0.3 mM Isopropyl β-d-1-thiogalactopyranoside (IPTG, Biosolve Chimie, France). Cells were lysed using a microfluidizer LM20 (Microfluidics, Westwood (MA), USA) at 20,000 psi, soluble depolymerase was recovered in the supernatant after ultracentrifugation in a Beckman Type 70Ti rotor at 126,000× g for 30 min. The depolymerase was purified from the supernatant using HisPur Ni-NTA Resin (Thermo Fisher Scientific, Rockford (IL), USA) agarose beads under gravity flow rates. The column was equilibrated in buffer A (10 mM imidazole-HCl, 0.3M NaCl, 50 mM NaPi, pH7.4), washed with 10-bed volumes of buffer B (25 mM imidazole-HCl, 0.3M NaCl, 50 mM NaPi, pH7.4), and eluted in 15 mL of buffer C (0.5M imidazole-HCl, 0.3M NaCl, 50 mM NaP, pH7.4). Purified protein was dialyzed in 50 mM NaPi pH 7.4, 0.150 mM NaCl. Purification steps were controlled by SDS-PAGE (Fig. 1B).

**TABLE 3:** Sequences of primers used for depolymerase cloning

Primer name	Sequence 5’−3’	Reference
HP_PWKp9B-Nde-F	ggaattccatatgggtttagttaagagcgtgtac	This study
HP_PWKp9B_Xho-R	tccgctcgagttatgcggctacagagccataag	This study

### Structure prediction

The structure of the depolymerase was predicted using Alphafold 3 (available at https://alphafoldserver.com/) as monomer, homodimer, homotrimer, and homotetramer (38). Prediction-aligned errors were analyzed to evaluate the confidence of the prediction. The top-ranked model of the trimeric structure was used as the most likely structure.

### Halo formation assay double-layered agar plate

The depolymerase activity of the purified recombinant protein was confirmed by formation of halos on double-layered agar plates. Briefly, bacteria (OD≈1) were diluted 1:100 into LB soft agar and poured onto LB agar plate. Recombinant depolymerase was serially diluted in PBS, and 5 µL drops were dispensed on the surface. After overnight incubation at 37°C, formation of halos was visually assessed. Assays were performed in biological triplicates.

### Phage plaque spot assay on double-layered agar plate

Phage plaque formation was evaluated by spot assay. Briefly, log phase bacteria (OD≈1) were diluted 1:100 into LB soft agar and poured onto LB agar plate. Phage was serially diluted in SM buffer (Cold Spring Harbor Lab protocols), and 5 µL drops were dispensed on the surface. After overnight incubation at 37°C, lytic plaques were counted. Assays were performed in biological triplicates.

### Survival and Percoll density gradient

Percoll density gradient were used to assess the presence or absence of the capsule that impacts bacteria buoyancy as previously described (39, 40). Bacteria were grown overnight in LB broth. Cultures were spun down at 7,800 rpm for 10 min at 4°C. Bacteria were resuspended in PBS (10 × smaller volume) and treated or not with 25 µg/mL of recombinant depolymerase. Tubes were incubated at room temperature (20–25°C) for 5 min, 2 h or 24 h. A 10 µL aliquot was serially diluted to count viable bacteria after treatment. The rest of the suspension was mixed 1:1 with 0.22 µm-filtered Percoll (Cytiva, Uppsala, Sweden) in a 1.5 mL tube and spun down at 20,000 g for 10 min at 4°C. Difference in buoyancy indicated a depolymerase activity of DpoK51-9B against the strain capsule type. Assays were performed in biological triplicates.

### Activity on other capsule types

Activity of DpoK51-9B was tested against a collection of *K. pneumoniae* strains harboring KL1, KL2, KL10, KL15, KL16, KL17, KL18, KL21, KL23, KL24, KL25, KL28, KL30, KL36, KL46, KL47, KL51, KL52, KL64, KL74, KL102, KL105, KL107, KL108, KL112, KL114, KL123, KL124, KL143, and KL151 as predicted by Kaptive from genomic data (23, 31). Percoll density gradients were performed before and after exposure to the depolymerase for 2 h at 37°C.

### Fluorescence exclusion microscopy

Strains were grown in LB in presence or absence of 2.5 µg/mL of recombinant depolymerase. A smear was prepared on a glass microscopy slide from a late log-phase culture (OD ≈2) in LB broth. Sample was air-dried for 5 min, 3 µL of 2 mg/mL fluorescein isothiocyanate (FITC)–dextran 2,000 kDa (FD2000S-100MG, Sigma-Aldrich, St. Louis, MO) was added on the smear and firmly covered with a glass slip. Fluorescence and phase-contrast images were acquired using an Alpha Plan-Apochromat 100×/1.46 Ph3 (UV) VIS-IR oil-immersion objective (Zeiss) on an Axio Imager M2 microscope (Zeiss), equipped with an Axiocam 305 mono camera (Zeiss) and operated via Zen Blue software v3.5 (Zeiss). Images were captured using the following exposure settings: phase contrast, 30  ms; GFP (488  nm), 100  ms. Image processing was performed using Zen Blue v3.5.

### Liquid culture infection

Bacterial growth curves were conducted in 24-well plates in Epoch2 (Biotek) plate reader at 37°C with continuous orbital shaking. *K. pneumoniae* strains were grown in LB at 37°C with shaking at 180  rpm until OD₆₀₀≈1. Cultures were diluted 1:100 in fresh buffered LB medium with calcium and magnesium, the depolymerase (250 ng/mL) and/or the phage added to a multiplicity of infection (MOI) of 0.1. OD_600nm_ was followed every 15 min during 18 h. Assays were performed in biological triplicates.

### Aggregation assay

Samples of the recombinant depolymerase were incubated for 30 min at the indicated temperature in a thermostatic dry block. Samples were separated into pellet and supernatant fractions by centrifugation in an Eppendorf microfuge (20,000× g, 5 min). The pellet (insoluble) fraction was washed once with reaction buffer. The supernatants were precipitated by addition of an equal volume of 15% trichloroacetic acid, centrifuged, and washed in 70% ethanol. Pellets were resuspended in SDS sample buffer. All reactions were resolved on SDS PAGE gels followed by staining with Coomassie Brilliant Blue. Assays were performed in biological triplicates.

### Activity following thermal aggregation

Aliquots of the recombinant depolymerase were incubated for 30 min at the indicated temperature in a thermostatic dry block. After cooling down, proteins were serially diluted 1/10 and was spotted on double-layered agar. Activity was confirmed by halo formation assay double-layered agar plate as described above. Assays were performed in biological triplicates.

### Thermal shift assay

The melting temperature of the recombinant depolymerase was determined by thermal shift assay (41). An aliquot of 45 μL of protein, concentrated to 1.5 mg/mL using Amicon 10 kDa, was mixed with Sypro Orange at 20× final concentration in a 96-well plate (Life Technologies, Eugene, OR, USA) as previously described (42, 43). Temperature was ramped up by 0.5°C increments, and fluorescence followed using the thermocycler Biorad CFX Connect Real-Time PCR Detection System. The melting temperature of the protein was defined as the maximum of the first-order derivative of the fluorescence emission as a function of temperature. Assays were performed in technical triplicates.

### GPH82 DNA extraction, sequencing, and assembly

Phage DNA extraction was performed as follows. Initially, the phage lysate was concentrated and washed with SM buffer using an Amicon 100 kDa centrifugal filter unit, and the concentrate was recovered in 250 µL of SM buffer. The concentrate was then treated with DNase at a concentration of 200 µg/mL for 2 h to remove any contaminating host DNA, followed by DNase inactivation using 15 mM EDTA with a subsequent incubation at 75°C for 10 minutes. To release the phage DNA, 10 µL of 10% SDS was added along with proteinase K (20 mg/mL), and the reaction mixture was incubated at 55°C for 45 min. Finally, the phage DNA was purified and concentrated using the DNA Clean and Concentrator kit (Zymo). Phage DNA sequencing was performed using Oxford Nanopore Technologies on a GridION instrument using the Rapid Barcoding Kit V14 (SQK-RBK114.96) following the manufacturer’s guidelines, then loaded onto an R10.4.1 flow cell. Following data acquisition, superaccuracy base calling was applied to ensure high-fidelity sequence determination. Phage genome assembly was performed within the Galaxy platform using Flye (Galaxy Version 2.9.5+galaxy1) with default parameters after a random read sub-sampling step that retained 5% of the reads via seq_tk resulting in a single circular contig of 111,710 bp with an average coverage of 127. Following the initial assembly, the contig was polished using the medaka consensus pipeline (Galaxy Version 1.7.2+galaxy1). The assembled phage GPH82 was assigned to the *Sugarlandvirus* genus using taxMyPhage (v3.3.6) available at https://ptax.ku.dk/ (44). BLAST analysis showed that the closest related phage was *Klebsiella* phage Sealy (accession number PQ337358.1) and revealed a 96% coverage with 95.38% identity.

## RESULTS

### Phage characterization and depolymerase prediction

The phage PWKp9B, previously isolated against the emerging *K. pneumoniae* ST16 clone by Martins et al. (33), formed clear lytic plaques surrounded by large turbid halos on bacterial lawns (Fig. 1A). These halos are a recognized hallmark of depolymerase activity and provide a convenient way to screen for phages with depolymerase able to degrade the bacterium’s thick polysaccharide capsule (20). These halos expanded over time when the plates were incubated at room temperature, further supporting enzymatic degradation of the capsule.

To investigate the genetic basis for this depolymerase activity, we next examined the phage genome for potential capsule-degrading enzymes. Genomic analysis previously revealed that PWKp9B is a 44.9 kb *Drulisvirus* belonging to the *Autoscriptoviridae* family, formerly known as *Autographiviridae*. To identify putative depolymerases, the phage sequence (Accession number: MZ634343.1 [33]) was analyzed using the depolymerase finder phageDPO tool, a machine learning–based predictor of depolymerase genes (36). Predicting depolymerases is challenging due to the low sequence homology among enzymes targeting different capsule types, which limits traditional homology-based approaches. We identified PWKp9B-orf2 (accession number: UJD05002.1), encoding a 787-amino-acid (84.7 kDa) protein, as a putative depolymerase, hereafter referred to as DpoK51-9B. InterPro domain analysis revealed two conserved folds: an N-terminal domain anchoring the spike protein to the phage tail (PFAM PF18668, AA 32–78) and a pectin lyase fold domain (IPR011050, AA 94–336), a structural feature often encountered in depolymerase structures, likely responsible for polysaccharide degradation (Fig. 1C) (20).

### Structural modeling and recombinant expression

Using AlphaFold 3 (38), we modeled the putative depolymerase as a monomer, dimer, trimer, and tetramer. Among these, the trimeric model exhibited the lowest predicted aligned error (PAE), as shown in the PAE plots (Fig. 1D), suggesting that the DpoK51-9B protein most likely adopts a trimeric conformation. This is consistent with previously characterized depolymerases, which commonly form trimeric structures (25, 26, 45). For further analyzes, we used the highest-scoring trimeric model. In contrast, the N-terminal domains were predicted with lower confidence, and their spatial orientation relative to the catalytic domain remained uncertain. The monomeric subunit adopts a right-handed β-helix fold, a structural motif commonly observed in capsule depolymerases.

To experimentally validate the function of the predicted depolymerase derived from phage PWKp9B, we next expressed and purified the recombinant protein. The gene encoding the putative recombinant depolymerase was cloned in pET24a with a C-terminal His-tag for purification. High-level expression of the recombinant protein was observed in the soluble fraction of the crude lysate following ultracentrifugation, indicating good solubility. The protein bound efficiently to Ni-NTA affinity columns and was eluted at 0.5 M imidazole, yielding a highly pure preparation (>95%) as assessed by SDS-PAGE (Fig. 1B).

### Enzymatic activity and capsule specificity

**Fig 2:**
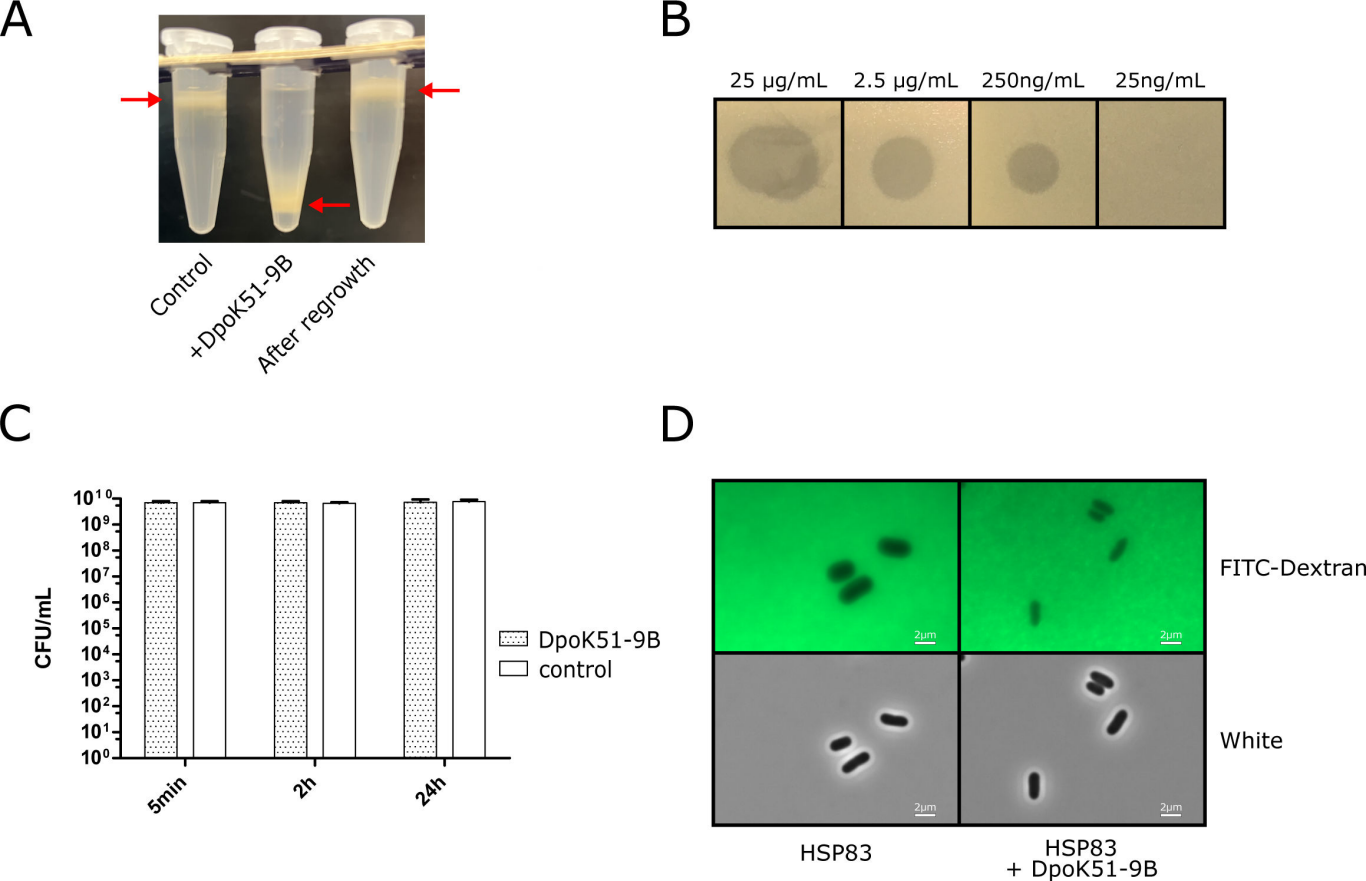
Activity of the recombinant depolymerase (**A**) Percoll density gradient showing increased density of *K. pneumoniae* cells following treatment with recombinant depolymerase. (**B**) Halo formation assay by the recombinant depolymerase on double-layered agar. (**C**) Bacterial survival (CFU/mL) following depolymerase treatment. (**D**) Fluorescence microscopy showing reduced FITC-dextran exclusion zone after depolymerase treatment.

We performed a series of assays to confirm the enzymatic activity and specificity of the recombinant depolymerase. First, in the continuous 50% Percoll density gradient assay, wild-type (WT) ST16 bacteria migrated to an upper position or floated, indicating the presence of an intact capsule. Upon treatment with the depolymerase, the bacteria shifted to the bottom of the tube, demonstrating an increase in bacterial density due to capsule degradation which occurred in less than 5 min (Fig. 2A). Upon regrowth of the bacteria in the absence of the enzyme, the strain regained its capsule, confirming that the depolymerase activity was reversible and did not result in permanent capsule loss. To evaluate potency, the purified depolymerase was spotted on double-layered agar, where it formed a halo, indicative of capsule degradation. Activity was detectable at concentrations as low as 250 ng/mL (2.95 nM), suggesting high enzymatic efficiency against the KL51 capsule (Fig. 2B). Importantly, no significant reduction in colony-forming units (CFU/mL) was observed following treatment (Fig. 2C), indicating that the enzyme specifically targets the capsule without bactericidal effects. Capsule visualization using FITC-labeled dextran, which highlights the polysaccharide exclusion zone, further supported these findings. A prominent exclusion zone was observed around untreated bacteria, which was dramatically diminished following depolymerase treatment (Fig. 2D).

Given the diversity of capsular types in *K. pneumoniae*, we evaluated the capsule specificity of the depolymerase. The depolymerase DpoK51-9B exhibited specific activity against *K. pneumoniae* strains carrying the KL51 capsular locus, including ST16 isolates from Brazil, France, and Switzerland, as well as ST231 isolates from Pakistan (Table 1). No depolymerase activity was observed on strains harboring other capsule types, including KL1, KL2, KL10, KL15, KL16, KL17, KL18, KL21, KL23, KL24, KL25, KL28, KL30, KL36, KL46, KL47, KL52, KL64, KL74, KL102, KL105, KL107, KL108, KL112, KL114, KL123, KL124, KL143, and KL151.

**Fig 3:**
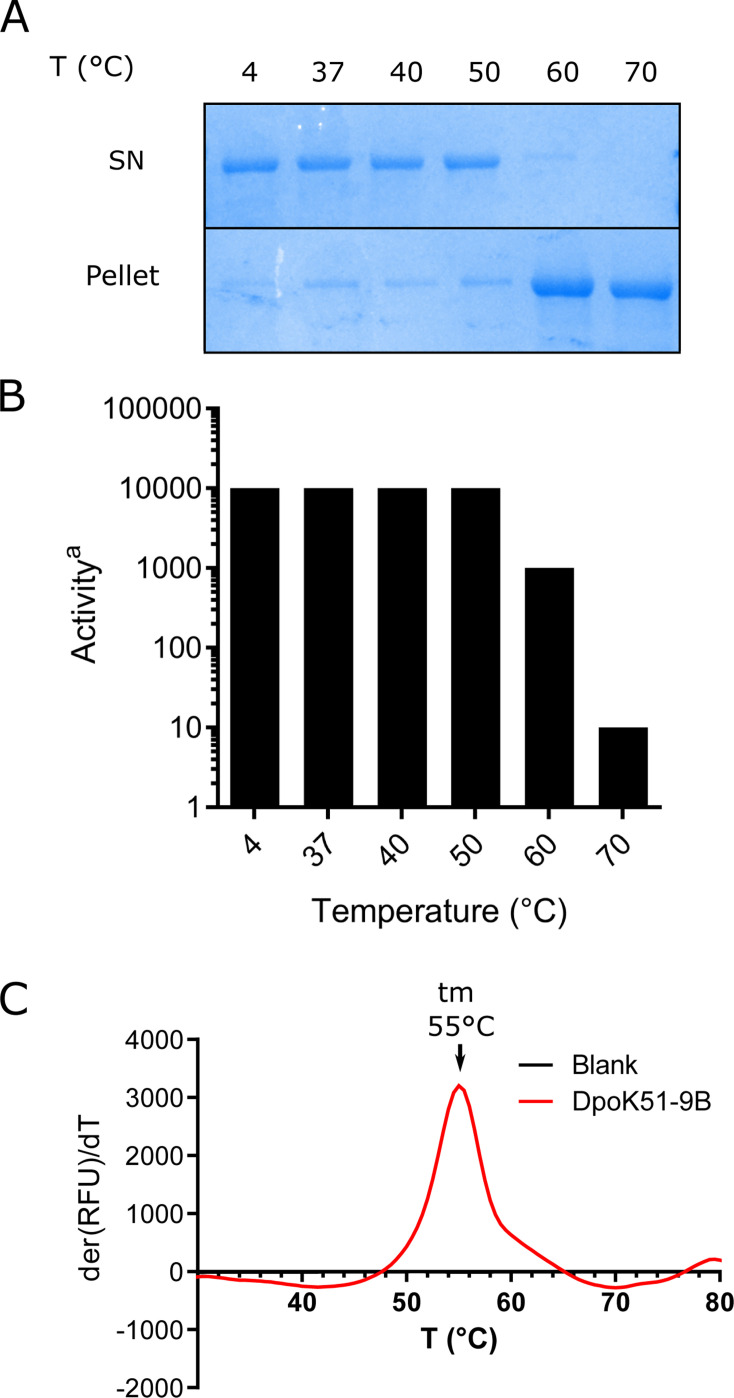
Depolymerase stability (**A**) Thermal aggregation assay of the recombinant DpoK51-9B. (**B**) Depolymerase activity, following incubation at various temperatures. Activity was quantified as the highest dilution showing a visible halo in a spot assay on double-layered agar. The assay was performed in triplicate with no observed variability. (**C**) Differential scanning fluorimetry of DpoK51-9B displayed as the first derivative of fluorescence units. The melting temperature (tm) was determined to be 55°C.

We next assessed the thermal stability of the enzyme to evaluate its robustness for potential therapeutic use. The recombinant depolymerase exhibited good thermal stability, maintaining solubility and enzymatic activity after 30 min of incubation at temperatures up to 50°C (Fig. 3A and B). At 60°C, the protein showed signs of aggregation and displayed approximately a 10-fold reduction in activity. Following exposure to 70°C, the protein was fully aggregated and exhibited a near-complete loss of activity, indicating a loss of structural integrity at higher temperatures. The melting temperature of the recombinant DpoK51-9B was measured at 55°C using differential scanning fluorimetry (Fig. 3C).

### Phage-depolymerase synergy and resistance analysis

**Fig 4:**
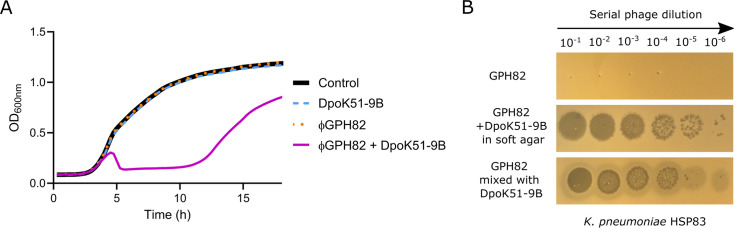
Synergistic activity of KL51 depolymerase DpoK51-9B and phage GPH82 against *K. pneumoniae* ST16 strain HSP83. (**A**) Liquid culture assay demonstrating synergistic activity leading to bacterial growth inhibition during 12 h by the combination of depolymerase and phage GPH82. Neither phage GPH82 nor depolymerase DpoK51-9B alone inhibited bacterial growth. Mean±SD (*n* = 3) are provided in Fig. S1 together with the treatment by phage pWKp9B as a control. (**B**) Synergy plaque formation assay showing lysis when recombinant depolymerase DpoK51-9B is combined with the phage GPH82.

We then assessed whether the depolymerase could enhance the antibacterial activity of phage GPH82, which is otherwise inactive against encapsulated ST16 strains. Liquid infection assays showed that neither the depolymerase DpoK51-9B nor the phage GPH82, when used individually, impacted the growth of the ST16 HSP83 isolate (Fig. 4A). However, their combination effectively suppressed bacterial growth for up to 12 h, after which regrowth of resistant bacteria was observed. This synergy, defined as the functional cooperation whereby capsule degradation by the depolymerase enables infection by the phage, was further supported by results from double-layer agar assays. In the presence of DpoK51-9B, either mixed directly with the phage or incorporated into the soft agar, the otherwise inactive phage GPH82 was able to form clear lytic plaques on strain HPS83 lawn (Fig. 4B).

To investigate potential resistance development, we analyzed bacteria that outgrew the phage-depolymerase combination *in vitro*. Bacteria that outgrew the combination in liquid culture were isolated from three independent experiments. Growth curve analyzes confirmed that these outgrowth clones were resistant to the phage-depolymerase combination. However, they remained encapsulated in the absence of the depolymerase and were still susceptible to capsule degradation by DpoK51-9B, in Percoll density gradient shifts and halo formation assays. Similar results were obtained from colonies isolated from the center of lytic spots on double-layer agar plates. These findings suggest that resistance was acquired specifically against phage GPH82, while susceptibility to the depolymerase was retained. The emergence of phage-resistant mutants is a common phenomenon *in vitro* and an important aspect to monitor in the context of phage therapy. This issue, however, is not specific to the phage/depolymerase combination tested in our study but applies broadly to phage-based approaches. Notably, across all experiments, no resistance to the depolymerase itself was observed following *in vitro* exposure to the phage-depolymerase combination.

### Comparison with other K51 depolymerases

**Fig 5:**
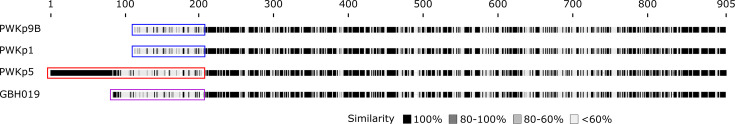
Protein sequence alignment of K51 depolymerases from different phages. Sequence similarity is represented by shading as visualized in Geneious Prime (2025.0.3) using the Blosum62 scoring matrix. The variable N-terminal region is highlighted with a colored box to emphasize sequence divergence in this domain.

To assess the sequence diversity and conservation among KL51-targeting depolymerases, we performed multiple protein sequence alignments (Fig. 5). The depolymerase DpoK51-9B, identified in phage PWKp9B, was compared with a previously reported KL51-targeting depolymerase encoded by the jumbo phage vB_KpnM_GBH019 (46). Additionally, using BLAST analysis, we identified two additional KL51-targeting depolymerases in anti-ST16 phages PWKp1 (DpoK51-1) and PWKp5 (DpoK51-5), which were isolated in the same study as PWKp9B (33). DpoK51-1 from phage PWKp1 was nearly identical to DpoK51-9B, sharing 98.3% amino acid identity across the full-length protein; both phages belonging to the *Drulivirus*. In contrast, DpoK51-5 from phage PWKp5 exhibited only 78% query coverage and 46.55% identity to DpoK51-9B, suggesting a more divergent enzyme. Notably, DpoK51-5 shared a higher degree of similarity with the depolymerase from phage vB_KpnM_GBH019, showing 81% coverage and 91.83% identity. Sequence and structure analysis revealed a bipartite domain architecture common to many phage depolymerase proteins, consistent with previous studies describing the modular organization of phage structural proteins (47). The N-terminal domains were divergent among the KL51-targeting depolymerases and are presumed to function as anchor domains responsible for attachment to the phage tail structure. In contrast, the catalytic domains, responsible for recognition and degradation of the capsular polysaccharide, were moderately to highly conserved, showing 60 to >99% amino acid similarity across the analyzed K51-depolymerases.

Recombinant forms of these two additional K51 depolymerases (DpoK51-1 and DpoK51-5) were cloned, expressed, and purified following the same protocols established for DpoK51-9B. All purified proteins demonstrated comparable K51 capsule-depolymerizing activity and synergistic effects with the phage GPH82 (Fig. S2).

## DISCUSSION

In this study, we successfully identified and purified a recombinant depolymerase from the anti-ST16 phage pWKp9B and demonstrated its ability to degrade the KL51 capsule of *K. pneumoniae*. Importantly, we showed that this depolymerase synergized with phage GPH82, which alone was inactive against encapsulated ST16 bacteria, enabling its lytic activity. GPH82 belongs to the broad-spectrum *Sugarlandviridae* phages, which have been shown to infect various *K. pneumoniae* lineages (48, 49), but have no intrinsic activity against encapsulated ST16-KL51 strains.

Our findings demonstrate that capsule depolymerases can extend phage host ranges by degrading the capsule barrier and exposing otherwise hidden surface receptors, such as outer membrane proteins or lipopolysaccharides, thereby enabling infection of previously resistant strains. This observation is consistent with prior reports showing that capsule-deficient strains are susceptible to a wider range of phages (30). By combining depolymerases with non-capsule-specific broad-host-range phages, we present a strategy to overcome capsule-mediated resistance and expand the arsenal of phages active against encapsulated *K. pneumoniae*. We further validated the specificity and reproducibility of this capsule-targeting strategy by purifying depolymerases from two additional KL51-targeting phages using the same protocol. These enzymes showed similar activity profiles to DpoK51-9B, reinforcing the robustness of our approach. We also observed that capsule degradation was reversible upon bacterial regrowth in the absence of depolymerase, suggesting that depolymerase treatment does not induce permanent alterations in capsule biosynthesis. This reversibility may reduce the selective pressure for resistance development. Consistent with this, no resistance to the depolymerase emerged during repeated *in vitro* exposure, either alone or in combination with phage. This aligns with previous reports showing that recombinant depolymerase does not trigger the selection of capsule-mediated resistance mechanism (50, 51). The recombinant depolymerase DpoK51-9B demonstrated good thermal stability, retaining solubility and enzymatic activity up to 50°C. This stability supports its potential use in therapeutic applications at physiological temperature and suggests resilience to moderate heat exposure during storage, formulation, and delivery.

This strategy addresses a key limitation of anti-*Klebsiella* phage therapy: most phages, including those encoding depolymerases, have narrow capsule-type-specific host ranges, further restricted by receptor availability and bacterial defense systems. As a result, large and diverse phage banks are typically required for therapeutic use. Combining recombinant depolymerases with broad-host-range phages lacking capsule-degrading activity could substantially reduce this need, while also offering practical advantages, as recombinant proteins are easier to produce, standardize, and obtain regulatory approval for than whole phages. Although depolymerases remain capsule-type specific, their effective host range is still broader than phages. For clinical application, panels of depolymerases would need to be assembled, but epidemiological studies show that only about a dozen capsule types dominate among hypervirulent and high-risk clones, suggesting that effective coverage is achievable.

While our study provides strong *in vitro* evidence of depolymerase-phage synergy, the broader applicability of this approach across other *K. pneumoniae* capsule types and phages remains to be validated. Notably, this strategy is likely effective only when combined with a capsule-independent phage. Further studies are needed to assess the efficacy of this approach *in vivo*, particularly in the context of infection models. In addition, future work should evaluate the safety, stability, delivery mechanisms, and clinical efficacy of phage-depolymerase combinations to fully realize their therapeutic potential.

## Data Availability

GPH82 sequence is available under NCBI Biosample SAMN49946575. The genome sequences of the anti-ST16 phages used in this study are available in GenBank under the following accession numbers: PWKp9B (MZ634343.1), PWKp5 (MZ634341.1), and PWKp1 (MZ634338.1).

## SUPPLEMENTAL MATERIAL

Figures S1 and S2 spectrum.02142-25-s0001.docx Figure S1: Growth curves with SD. Figure S2: Data for additional K51 depolymerases.

ASM does not own the copyrights to Supplemental Material that may be linked to, or accessed through, an article. The authors have granted ASM a non-exclusive, world-wide license to publish the Supplemental Material files. Please contact the corresponding author directly for reuse.

## References

[1] World Heath Organization. 2024. WHO bacterial priority pathogens list, 2024: bacterial pathogens of public health importance to guide research, development and strategies to prevent and control antimicrobial resistance. 10.1016/S1473-3099(25)00118-5 PMC12367593 40245910

[2] Murray CJL, Ikuta KS, Sharara F, Swetschinski L, Robles Aguilar G, Gray A, Han C, Bisignano C, Rao P, Wool E, et al.. 2022. Global burden of bacterial antimicrobial resistance in 2019: a systematic analysis. Lancet 399:629–655. doi:10.1016/S0140-6736(21)02724-0 35065702 PMC8841637

[3] Miller WR, Arias CA. 2024. ESKAPE pathogens: antimicrobial resistance, epidemiology, clinical impact and therapeutics. Nat Rev Microbiol 22:598–616. doi:10.1038/s41579-024-01054-w 38831030 PMC13147291

[4] Baquero F, Coque TM. 2011. Multilevel population genetics in antibiotic resistance. FEMS Microbiol Rev 35:705–706. doi:10.1111/j.1574-6976.2011.00293.x 21714793

[5] Abe R, Akeda Y, Takeuchi D, Sakamoto N, Sugawara Y, Yamamoto N, Kerdsin A, Matsumoto Y, Motooka D, Leolerd W, Santanirand P, Suzuki M, Shibayama K, Tomono K, Iida T, Hamada S. 2022. Clonal dissemination of carbapenem-resistant Klebsiella pneumoniae ST16 co-producing NDM-1 and OXA-232 in Thailand. JAC-Antimicrobial Resistance 4:dlac084. doi:10.1093/jacamr/dlac084 35983103 PMC9380991

[6] Andrey DO, Pereira Dantas P, Martins WBS, Marques De Carvalho F, Almeida LGP, Sands K, Portal E, Sauser J, Cayô R, Nicolas MF, Vasconcelos ATR, Medeiros EA, Walsh TR, Gales AC. 2020. An emerging clone, Klebsiella pneumoniae carbapenemase 2-producing K. pneumoniae sequence type 16, associated with high mortality rates in a CC258-endemic setting. Clin Infect Dis 71:e141–e150. doi:10.1093/cid/ciz1095 31712802 PMC7583420

[7] Hennequin C, Chlilek A, Beyrouthy R, Bonnet R, Robin F. 2018. Diversity of DHA-1-encoding plasmids in Klebsiella pneumoniae isolates from 16 French hospitals. J Antimicrob Chemother 73:2981–2989. doi:10.1093/jac/dky285 30060165

[8] Nguyen TNT, Nguyen PLN, Le NTQ, Nguyen LPH, Duong TB, Ho NDT, Nguyen QPN, Pham TD, Tran AT, The HC, Nguyen HH, Nguyen CVV, Thwaites GE, Rabaa MA, Pham DT. 2021. Emerging carbapenem-resistant Klebsiella pneumoniae sequence type 16 causing multiple outbreaks in a tertiary hospital in southern Vietnam. Microb Genom 7:mgen000519. doi:10.1099/mgen.0.000519 33565955 PMC8190610

[9] Sierra R, Roch M, Moraz M, Prados J, Vuilleumier N, Emonet S, Andrey DO. 2024. Contributions of long-read sequencing for the detection of antimicrobial resistance. Pathogens 13:730. doi:10.3390/pathogens13090730 39338921 PMC11434816

[10] Zhang B, Hu R, Liang Q, Liang S, Li Q, Bai J, Ding M, Zhang F, Zhou Y. 2022. Comparison of two distinct subpopulations of Klebsiella pneumoniae ST16 co-occurring in a single patient. Microbiol Spectr 10:e0262421. doi:10.1128/spectrum.02624-21 35467408 PMC9241866

[11] de Sales RO, Leaden L, Migliorini LB, Severino P. 2022. A comprehensive genomic analysis of the emergent Klebsiella pneumoniae ST16 lineage: virulence, antimicrobial resistance and a comparison with the clinically relevant ST11 strain. Pathogens 11:1394. doi:10.3390/pathogens11121394 36558729 PMC9781218

[12] Roch M, Sierra R, Sands K, Martins WMBS, Schrenzel J, Walsh TR, Gales AC, Andrey DO. 2021. Vertical and horizontal dissemination of an IncC plasmid harbouring rmtB 16S rRNA methylase gene, conferring resistance to plazomicin, among invasive ST258 and ST16 KPC-producing Klebsiella pneumoniae. J Glob Antimicrob Resist 24:183–189. doi:10.1016/j.jgar.2020.12.006 33373732

[13] Hatfull GF, Dedrick RM, Schooley RT. 2022. Phage therapy for antibiotic-resistant bacterial infections. Annu Rev Med 73:197–211. doi:10.1146/annurev-med-080219-122208 34428079

[14] Strathdee SA, Hatfull GF, Mutalik VK, Schooley RT. 2023. Phage therapy: from biological mechanisms to future directions. Cell 186:17–31. doi:10.1016/j.cell.2022.11.017 36608652 PMC9827498

[15] Herridge WP, Shibu P, O’Shea J, Brook TC, Hoyles L. 2020. Bacteriophages of Klebsiella spp., their diversity and potential therapeutic uses. J Med Microbiol 69:176–194. doi:10.1099/jmm.0.001141 31976857 PMC7431098

[16] Rendueles O. 2020. Deciphering the role of the capsule of Klebsiella pneumoniae during pathogenesis: a cautionary tale. Mol Microbiol 113:883–888. doi:10.1111/mmi.14474 31997409 PMC7317218

[17] Paczosa MK, Mecsas J. 2016. Klebsiella pneumoniae: going on the offense with a strong defense. Microbiol Mol Biol Rev 80:629–661. doi:10.1128/MMBR.00078-15 27307579 PMC4981674

[18] Beamud B, García-González N, Gómez-Ortega M, González-Candelas F, Domingo-Calap P, Sanjuan R. 2023. Genetic determinants of host tropism in Klebsiella phages. Cell Rep 42:112048. doi:10.1016/j.celrep.2023.112048 36753420 PMC9989827

[19] Haudiquet M, Le Bris J, Nucci A, Bonnin RA, Domingo-Calap P, Rocha EPC, Rendueles O. 2024. Capsules and their traits shape phage susceptibility and plasmid conjugation efficiency. Nat Commun 15:2032. doi:10.1038/s41467-024-46147-5 38448399 PMC10918111

[20] Knecht LE, Veljkovic M, Fieseler L. 2019. Diversity and function of phage encoded depolymerases. Front Microbiol 10:2949. doi:10.3389/fmicb.2019.02949 31998258 PMC6966330

[21] Pires DP, Oliveira H, Melo LDR, Sillankorva S, Azeredo J. 2016. Bacteriophage-encoded depolymerases: their diversity and biotechnological applications. Appl Microbiol Biotechnol 100:2141–2151. doi:10.1007/s00253-015-7247-0 26767986

[22] Wyres KL, Wick RR, Gorrie C, Jenney A, Follador R, Thomson NR, Holt KE. 2016. Identification of Klebsiella capsule synthesis loci from whole genome data. Microb Genom 2:e000102. doi:10.1099/mgen.0.000102 28348840 PMC5359410

[23] Lam MMC, Wick RR, Judd LM, Holt KE, Wyres KL. 2022. Kaptive 2.0: updated capsule and lipopolysaccharide locus typing for the Klebsiella pneumoniae species complex. Microb Genom 8:000800. doi:10.1099/mgen.0.000800 35311639 PMC9176290

[24] Jiao X, Wang M, Liu Y, Yang S, Yu Q, Qiao J. 2025. Bacteriophage-derived depolymerase: a review on prospective antibacterial agents to combat Klebsiella pneumoniae. Arch Virol 170:70. doi:10.1007/s00705-025-06257-x 40057622

[25] Latka A, Leiman PG, Drulis-Kawa Z, Briers Y. 2019. Modeling the architecture of depolymerase-containing receptor binding proteins in Klebsiella phages. Front Microbiol 10:2649. doi:10.3389/fmicb.2019.02649 31803168 PMC6872550

[26] Cai R, Ren Z, Zhao R, Lu Y, Wang X, Guo Z, Song J, Xiang W, Du R, Zhang X, Han W, Ru H, Gu J. 2023. Structural biology and functional features of phage-derived depolymerase Depo32 on Klebsiella pneumoniae with K2 serotype capsular polysaccharides. Microbiol Spectr 11:e0530422. doi:10.1128/spectrum.05304-22 37750730 PMC10581125

[27] Dunstan RA, Bamert RS, Belousoff MJ, Short FL, Barlow CK, Pickard DJ, Wilksch JJ, Schittenhelm RB, Strugnell RA, Dougan G, Lithgow T. 2021. Mechanistic insights into the capsule-targeting depolymerase from a Klebsiella pneumoniae bacteriophage. Microbiol Spectr 9:e0102321. doi:10.1128/spectrum.01023-21 34431721 PMC8552709

[28] Bansal S, Harjai K, Chhibber S. 2014. Depolymerase improves gentamicin efficacy during Klebsiella pneumoniae induced murine infection. BMC Infect Dis 14:456. doi:10.1186/1471-2334-14-456 25149315 PMC4150946

[29] Wang C, Li P, Niu W, Yuan X, Liu H, Huang Y, An X, Fan H, Zhangxiang L, Mi L, Zheng J, Liu Y, Tong Y, Mi Z, Bai C. 2019. Protective and therapeutic application of the depolymerase derived from a novel KN1 genotype of Klebsiella pneumoniae bacteriophage in mice. Res Microbiol 170:156–164. doi:10.1016/j.resmic.2019.01.003 30716390

[30] Lourenço M, Osbelt L, Passet V, Gravey F, Megrian D, Strowig T, Rodrigues C, Brisse S. 2023. Phages against noncapsulated Klebsiella pneumoniae: broader host range, slower resistance. Microbiol Spectr 11:e0481222. doi:10.1128/spectrum.04812-22 37338376 PMC10433977

[31] Wick RR, Heinz E, Holt KE, Wyres KL. 2018. Kaptive web: user-friendly capsule and lipopolysaccharide serotype prediction for Klebsiella genomes. J Clin Microbiol 56:e00197-18. doi:10.1128/JCM.00197-18 29618504 PMC5971559

[32] Lam MMC, Wick RR, Watts SC, Cerdeira LT, Wyres KL, Holt KE. 2021. A genomic surveillance framework and genotyping tool for Klebsiella pneumoniae and its related species complex. Nat Commun 12:4188. doi:10.1038/s41467-021-24448-3 34234121 PMC8263825

[33] Martins W, Cino J, Lenzi MH, Sands K, Portal E, Hassan B, Dantas PP, Migliavacca R, Medeiros EA, Gales AC, Toleman MA. 2022. Diversity of lytic bacteriophages against XDR Klebsiella pneumoniae sequence type 16 recovered from sewage samples in different parts of the world. Sci Total Environ 839:156074. doi:10.1016/j.scitotenv.2022.156074 35623509

[34] Hassan B, Ijaz M, Khan A, Sands K, Serfas GI, Clayfield L, El-Bouseary MM, Lai G, Portal E, Khan A, Watkins WJ, Parkhill J, Walsh TR. 2021. A role for arthropods as vectors of multidrug-resistant Enterobacterales in surgical site infections from South Asia. Nat Microbiol 6:1259–1270. doi:10.1038/s41564-021-00965-1 34580444

[35] Sierra R, Roch M, Jaquier D, Andrey DO. 2022. The GENeva PHage (GENPH) collection against multidrug resistant Klebsiella pneumoniae, abstr viruses of microbes, Guimaraes, Portugal.

[36] Vieira M, Duarte J, Domingues R, Oliveira H, Dias O. 2023. PhageDPO: phage depolymerase finder. bioRxiv. doi:10.1101/2023.02.24.529883 39951981

[37] Blum M, Chang HY, Chuguransky S, Grego T, Kandasaamy S, Mitchell A, Nuka G, Paysan-Lafosse T, Qureshi M, Raj S, et al.. 2021. The InterPro protein families and domains database: 20 years on. Nucleic Acids Res 49:D344–D354. doi:10.1093/nar/gkaa977 33156333 PMC7778928

[38] Abramson J, Adler J, Dunger J, Evans R, Green T, Pritzel A, Ronneberger O, Willmore L, Ballard AJ, Bambrick J, et al.. 2024. Accurate structure prediction of biomolecular interactions with AlphaFold 3. Nature 630:493–500. doi:10.1038/s41586-024-07487-w 38718835 PMC11168924

[39] Ardissone S, Fumeaux C, Bergé M, Beaussart A, Théraulaz L, Radhakrishnan SK, Dufrêne YF, Viollier PH. 2014. Cell cycle constraints on capsulation and bacteriophage susceptibility. eLife 3:e03587. doi:10.7554/eLife.03587 25421297 PMC4241560

[40] Dorman MJ, Feltwell T, Goulding DA, Parkhill J, Short FL. 2018. The capsule regulatory network of Klebsiella pneumoniae defined by density-TraDISort. mBio 9:e01863-18. doi:10.1128/mBio.01863-18 30459193 PMC6247091

[41] Pinz S, Doskocil E, Seufert W. 2022. Thermofluor-based analysis of protein integrity and ligand interactions. Methods Mol Biol 2533:247–257. doi:10.1007/978-1-0716-2501-9_15 35796993 PMC9761908

[42] Huynh K, Partch CL. 2015. Analysis of protein stability and ligand interactions by thermal shift assay. Curr Protoc Protein Sci 79:28. doi:10.1002/0471140864.ps2809s79 PMC4332540 25640896

[43] Roch M, Lelong E, Panasenko OO, Sierra R, Renzoni A, Kelley WL. 2019. Thermosensitive PBP2a requires extracellular folding factors PrsA and HtrA1 for Staphylococcus aureus MRSA β-lactam resistance. Commun Biol 2:417. doi:10.1038/s42003-019-0667-0 31754647 PMC6858329

[44] Millard A, Denise R, Lestido M, Thomas MT, Webster D, Turner D, Sicheritz-Pontén T. 2025. taxMyPhage: automated taxonomy of dsDNA phage genomes at the genus and species level. Phage (New Rochelle) 6:5–11. doi:10.1089/phage.2024.0050 40351403 PMC12060842

[45] Squeglia F, Maciejewska B, Łątka A, Ruggiero A, Briers Y, Drulis-Kawa Z, Berisio R. 2020. Structural and functional studies of a Klebsiella phage capsule depolymerase tailspike: mechanistic insights into capsular degradation. Structure 28:613–624. doi:10.1016/j.str.2020.04.015 32386574

[46] Blundell-Hunter G, Enright MC, Negus D, Dorman MJ, Beecham GE, Pickard DJ, Wintachai P, Voravuthikunchai SP, Thomson NR, Taylor PW. 2021. Characterisation of bacteriophage-encoded depolymerases selective for key Klebsiella pneumoniae capsular exopolysaccharides. Front Cell Infect Microbiol 11:686090. doi:10.3389/fcimb.2021.686090 34222050 PMC8253255

[47] Latka A, Lemire S, Grimon D, Dams D, Maciejewska B, Lu T, Drulis-Kawa Z, Briers Y. 2021. Engineering the modular receptor-binding proteins of Klebsiella phages switches their capsule serotype specificity. mBio 12:e00455-21. doi:10.1128/mBio.00455-21 33947754 PMC8262889

[48] Concha-Eloko R, Barberán-Martínez P, Sanjuán R, Domingo-Calap P. 2023. Broad-range capsule-dependent lytic Sugarlandvirus against Klebsiella sp. Microbiol Spectr 11:e0429822. doi:10.1128/spectrum.04298-22 37882584 PMC10714931

[49] Townsend EM, Kelly L, Gannon L, Muscatt G, Dunstan R, Michniewski S, Sapkota H, Kiljunen SJ, Kolsi A, Skurnik M, Lithgow T, Millard AD, Jameson E. 2021. Isolation and characterization of Klebsiella phages for phage therapy. Phage (New Rochelle) 2:26–42. doi:10.1089/phage.2020.0046 33796863 PMC8006926

[50] Li P, Guo G, Zheng X, Xu S, Zhou Y, Qin X, Hu Z, Yu Y, Tan Z, Ma J, Chen L, Zhang W. 2024. Therapeutic efficacy of a K5-specific phage and depolymerase against Klebsiella pneumoniae in a mouse model of infection. Vet Res 55:59. doi:10.1186/s13567-024-01311-z 38715095 PMC11077817

[51] Islam MM, Mahbub NU, Shin WS, Oh MH. 2024. Phage-encoded depolymerases as a strategy for combating multidrug-resistant Acinetobacter baumannii. Front Cell Infect Microbiol 14:1462620. doi:10.3389/fcimb.2024.1462620 39512587 PMC11540826

