# Supplementary material for: Synergistic activity of a KL51-depolymerase and a Sugarlandvirus bacteriophage against ST16 Klebsiella pneumoniae
Source: Microbiol Spectr. 2025 Oct 27;13(12):e02142-25. doi: 10.1128/spectrum.02142-25 (PMC12671143; doi:10.1128/spectrum.02142-25)
Supplement: Figures S1 and S2 — Figure S1: Growth curves with SD. Figure S2: Data for additional K51 depolymerases. [file spectrum.02142-25-s0001.docx]

**Supplementary material**


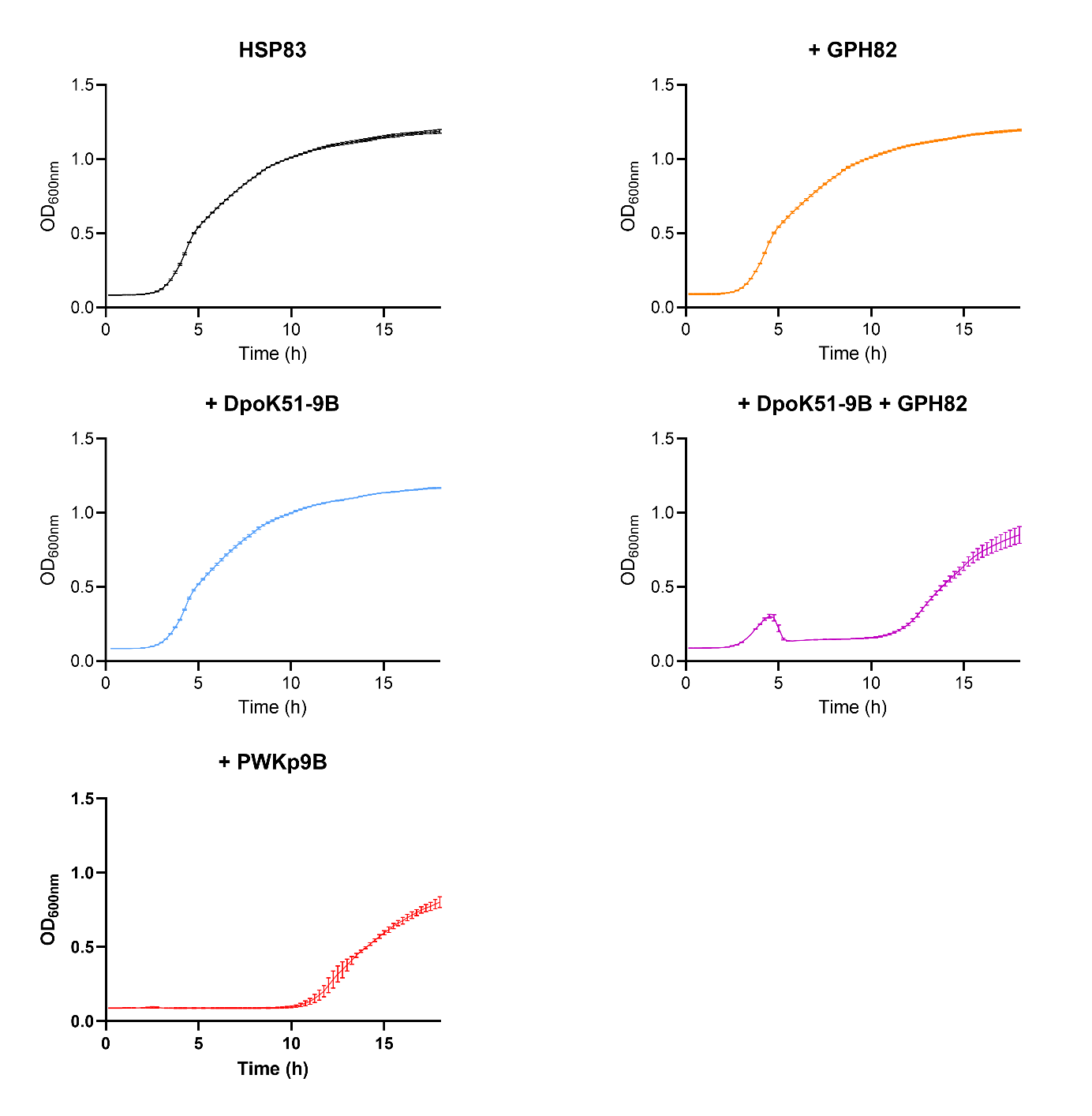


**Supplementary Figure 1**: Liquid culture assay (mean ±SD, n=3) demonstrating synergistic activity leading to bacterial growth inhibition during 12h by the combination of depolymerase and phage GPH82. Neither phage GPH82 nor depolymerase DpoK51-9B alone inhibited bacterial growth. Phage PKp9B harboring the depolymerase is display as control.


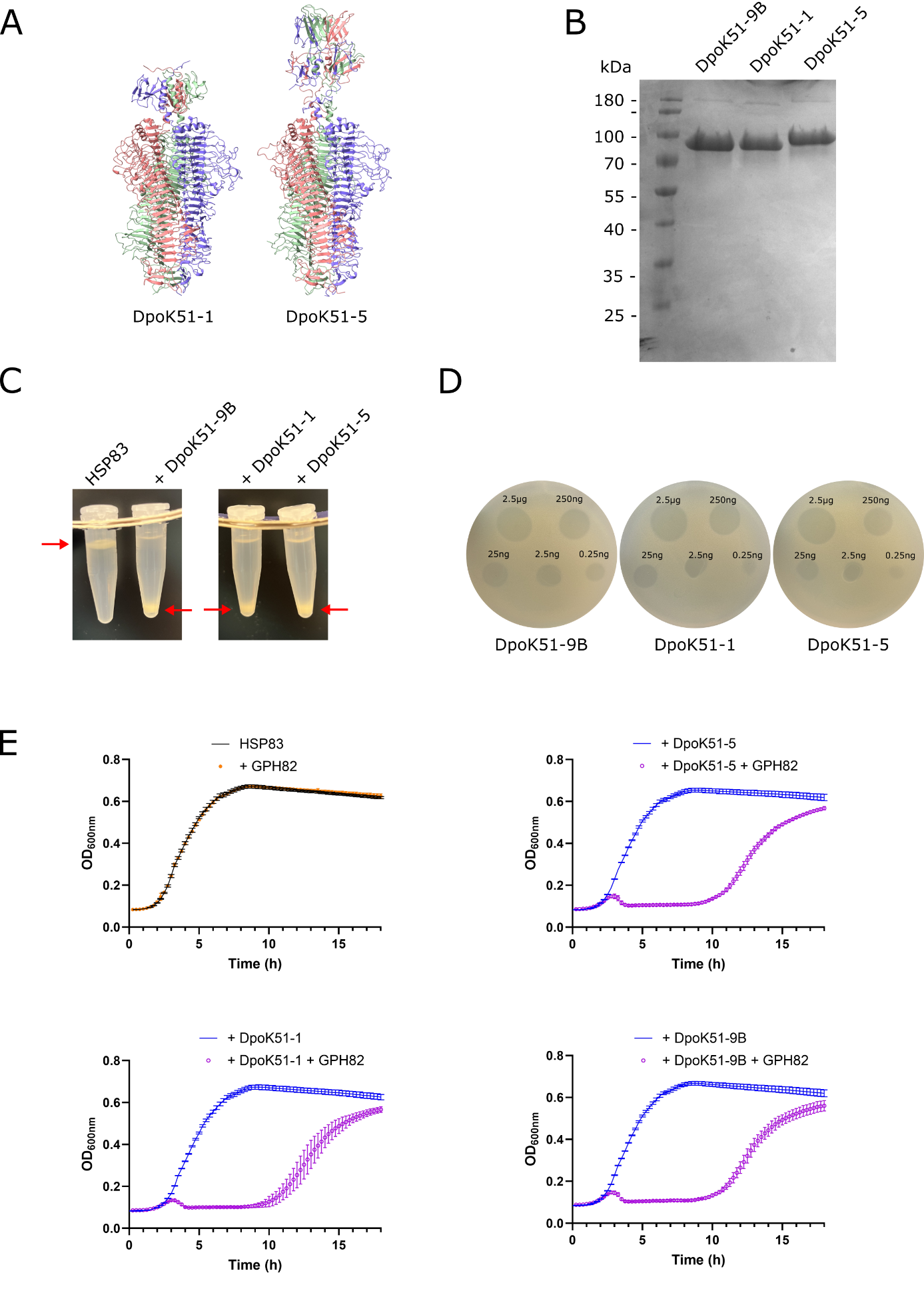
**Supplementary Figure 2:** (A) AlphaFold-predicted structure of the depolymerase Dpo-K51-1 and DpoK51-5 (B) SDS-PAGE gel stained with Coomassie Brilliant Blue showing the purity of the recombinant K51 depolymerases (C) Percoll density gradient showing decreased buoyancy of *K. pneumoniae* cells following treatment with recombinant depolymerase. (D) Halo formation assay by the recombinant depolymerase on double-layered agar. (E) Liquid culture assay demonstrating synergistic activity leading to bacterial growth inhibition by the combination of K51 depolymerases and phage GPH82.
